# Natural language processing in at-risk mental states: enhancing the assessment of thought disorders and psychotic traits with semantic dynamics and graph theory

**DOI:** 10.47626/1516-4446-2023-3419

**Published:** 2024-11-25

**Authors:** Felipe Argolo, William Henrique de Paula Ramos, Natalia Bezerra Mota, João Medrado Gondim, Ana Caroline Lopes-Rocha, Julio Cesar Andrade, Martinus Theodorus van de Bilt, Leonardo Peroni de Jesus, Andrea Jafet, Guillermo Cecchi, Wagner Farid Gattaz, Cheryl Mary Corcoran, Anderson Ara, Alexandre Andrade Loch

**Affiliations:** 1 Laboratório de Neurociências (LIM 27), Instituto de Psiquiatria, Hospital das Clínicas, Faculdade de Medicina, Universidade de São Paulo, São Paulo, SP, Brazil; 2 Departamento de Estatística, Universidade Federal do Paraná, Curitiba, PR, Brazil; 3 Instituto de Psiquiatria, Departamento de Psiquiatria e Medicina Legal, Universidade Federal do Rio de Janeiro, Rio de Janeiro, RJ, Brazil; 4 Research Department at Motrix Lab, Rio de Janeiro, RJ, Brazil; 5 Instituto de Computação, Universidade Federal da Bahia, Salvador, BA, Brazil; 6 Instituto Nacional de Biomarcadores em Neuropsiquiatria, Conselho Nacional de Desenvolvimento Científico e Tecnológico, Brazil; 7 IBM Thomas J. Watson Research Center, Yorktown Heights, NY, USA; 8 Icahn School of Medicine at Mount Sinai, New York, NY, USA; 9 James J. Peters Department of Veterans Affairs Medical Center, Bronx, NY, USA

**Keywords:** Psychosis, at-risk mental states, natural language processing, semantics, screening, machine learning

## Abstract

**Objective:**

Verbal communication contains key information for mental health assessment. Researchers have linked psychopathology phenomena to certain counterparts in natural language processing. We characterized subtle impairments in the early stages of psychosis, developing new analysis techniques, which led to a comprehensive map associating features of natural language processing with the full range of clinical presentation.

**Methods:**

We used natural language processing to assess spontaneous and elicited speech by 60 individuals with at-risk mental states and 73 controls who were screened from 4,500 quota-sampled Portuguese speaking residents of São Paulo, Brazil. Psychotic symptoms were independently assessed with the Structured Interview for Psychosis-Risk Syndromes. Speech features (e.g., sentiments and semantic coherence), including novel ones, were correlated with psychotic traits (Spearman’s-ρ) and at-risk mental state status (general linear models and machine-learning ensembles).

**Results:**

Natural language processing features were informative for classification, presenting a balanced accuracy of 86%. Features such as semantic laminarity (as perseveration), semantic recurrence time (as circumstantiality), and average centrality in word repetition graphs carried the most information and were directly correlated with psychotic symptoms. Grammatical tagging (e.g., use of adjectives) was the most relevant standard measure.

**Conclusion:**

Subtle speech impairments can be detected by sensitive methods and can be used in at-risk mental states screening. We have outlined a blueprint for speech-based evaluation, pairing features to standard psychometric items for thought disorder.

## Introduction

Speech structure and meaning have long been used to identify underlying processes of human cognition.^1^-^3^ In mental health care, speech patterns have been studied using natural language processing (NLP) during the last decade. NLP helps researchers formulate models that correspond to observable psychopathology phenomena and then assess cross-validity between NLP features and standard psychometric evaluations.

Accurate description of abstract alterations in the structure of verbal expression can improve the characterization of mental impairment. Such information can increase treatment effectiveness by improving diagnosis and assessment, allowing for early intervention.^4^

Our study replicates results that demonstrate experimental associations between language patterns and psychotic symptoms in individuals with at-risk mental states (ARMS). We introduce concepts from dynamic systems theory (recurrence quantification analysis [RQA]) and graph theory (indirect connections between “friends-of-friends” using centrality) that contribute to a comprehensive scheme linking clinical taxonomy and NLP vocabulary.

Prototypical alterations in spoken language can be found in psychosis, an early entity in psychiatry historically associated with impairments in abstract thinking and speech production (DeLisi^5^). In fact, the concepts of psychosis and thought disorder are intertwined.^6^ Psychotic speech presents both structural (e.g., strained syntax, word repetitions) and semantic (e.g., thought derailment) alterations, requiring different approaches to characterize it.^7^-^12^ Over the last decade, numerous studies have been conducted to operationalize speech impairments in psychosis and its risk states using NLP.^10^,^13^,^14^

The ARMS framework has been used in the early detection and prevention of psychosis for almost three decades.^15^ ARMS comprises individuals with subclinical psychotic phenomena who do not yet have the disorder but are at short-term risk of threshold psychosis. Compared to the changes observed in diagnosed disorders, such as word salad in severe schizophrenia, speech characteristics of ARMS are generally more subtle.^9^ Nevertheless, they are still detectable and affect a number of features, including semantic coherence, word repetition patterns, and tangentiality.^16^

NLP formalizes the analysis of spoken language, rigorously characterizing features of human speech. It usually involves computerized tools and statistical inference.^14^,^17^ For instance, poverty of speech, seen in both schizophrenia and ARMS, can be simply understood as the total number of words uttered or the average phrase size. The loose association of ideas and abrupt changes of topic (e.g., derailment) seen in the psychosis spectrum are modeled as large distances between words in latent semantic spaces.^18^

Semantics and structure can be considered two intersecting facets of speech. In semantics analysis, researchers often observe similarity between the meaning of words (coherence) and associations with human affect (e.g., positive and negative feelings). Structural analysis generally targets syntactic functions^19^ and word repetition, as seen in previous psychosis studies.^18^,^20^

The semantic similarity of two words can be assessed by calculating their relative distance in abstract spaces. For instance, “blue” and “purple” are expected to be closer than “rocket” and “frog.” This type of analysis was pioneered in the latent semantic analysis technique,^21^ in which discourse is represented in mathematically tractable spaces as vectors. We often estimate distances by taking the cosine similarity of the angle between vectors representing two words.^22^ Measures of semantic similarity are also markedly different in psychotic speech.^23^ First order coherences (FOC) (similarity of consecutive words) and second order coherences (SOC) (similarity of two nearly consecutive words, i.e., separated by a single word) are often studied as features.^13^,^24^ They allude to the classic clinical description of derailment: jumping from one theme to an unrelated idea.

In semantic analysis of sentiments, each text segment is classified according to a previous reference guide that classifies words with respect to affective domains (e.g., positive and negative). It is regularly used in social media to understand public reaction to a given topic.^25^ It has also been reported as an informative feature of clinical status in psychosis.^9^

Grammatical tagging consists of parsing sentences and labeling words according to their syntactic function. The relative frequency of each class and other related features are used to describe speech characteristics. Part-of-speech tagging suggests that certain grammatical classes are used differently among psychotic individuals. Specifically, independent studies have detected differences in adjective and pronoun frequency between patients and healthy controls and in psychosis onset in at-risk individuals.^13^,^26^

Another NLP technique for structural analysis involves representations based on graph theory. In word repetition graphs,^27^,^28^ symbols are associated with nodes in a graph and lines are drawn for every consecutive appearance between two words. Some properties, such as connectivity, are known to vary between people with and without mental illness.^18^ Commonly, we assess connectivity through the number and size of closed loops in speech (e.g., strongly connected components [SCC]). In the psychotic spectrum, the less connected the speech, the more severe the negative symptoms are at different stages.^28^,^29^

To conclude, important advances have been made in language analysis in psychosis. Nevertheless, research on subthreshold presentations such as ARMS, which might present subtle and heterogeneous signals, is still scarce. Moreover, classical aspects of thought, speech, and communication disorders (e.g., perseveration) have not been modeled. A comprehensive scheme connecting clinical taxonomy and NLP vocabulary has yet to be constructed.

Our study replicates experimental findings of associations between language patterns (NLP features), psychotic symptoms (negative, positive, disorganized, and general symptoms), and ARMS status. To address the remaining gaps in clinical feature modeling, we leveraged procedures from dynamical systems (RQA) and graph theory (indirect connections between “friends-of-friends” using centrality).

Our findings are derived from a sample of 133 non-help-seeking individuals (60 with ARMS and 73 controls) screened out of 4,500 randomly selected residents of São Paulo, Brazil, the most populous city in South America.

Our main hypothesis was that standard classic NLP features would be associated with ARMS status and specific psychopathology. We also expected some new NLP features to be associated with the same clinical constructs. Regarding structure, we tested for 1) the frequency of words from a certain grammatical class and 2) structural properties derived from graph embeddings, including centrality (see the Methods section for a detailed description of all measures). Concerning semantics, we analyzed 3) coherence measures, including RQA, and 4) valence (e.g., positive and negative sentiments) in speech. These measures were correlated with symptoms (Structured Interview for Psychosis-Risk Syndromes [SIPS]) and overall ARMS status.

Finally, we will discuss an overall blueprint connecting clinically described phenomena in psychopathology and formal NLP-based models.

## Methods

### Sampling and screening

This study is part of the Subclinical Symptoms and Prodromal Psychosis Project, a population-based cohort study in São Paulo, SP, Brazil involving 4,500 Portuguese-speaking individuals aged 18-35 years.^30^ First, individuals were interviewed by telephone or face-to-face using the Prodromal Questionnaire-Brief version^31^ and the Basic Symptoms Scale, following previously published screening procedures.^32^ A shorter version of the original 92-item Prodromal Questionnaire,^33^ this self-report instrument screens ARMS for psychosis through 16 items on positive psychotic symptoms.^31^ The Basic Symptoms Scale assesses symptoms of self-experienced perception and cognition disturbances present in the initial manifestations of psychosis risk.^32^,^34^ Individuals with a combined score > 10 on the Prodromal Questionnaire-Brief version+ Basic Symptoms Scale were invited for face-to-face interviews at the Universidade de São Paulo’s Instituto de Psiquitaria.

### Structured interviews

Experienced psychiatrists assessed participants with the SIPS^35^,^36^ for ARMS status and the Structured Clinical Interview for DSM-5.^37^ The SIPS is a 19-item scale divided into four symptom domains (positive, negative, disorganization, general). The instrument also includes the Global Assessment of Functioning Scale, an assessment of schizotypal personality disorder criteria, a questionnaire on family history of mental illness, and two operational definitions (criteria for prodromal syndromes and criteria for psychotic syndrome), which are used to determine the three prodromal syndromes and diagnosable psychosis.^36^,^38^ The syndromes include brief intermittent psychotic symptom syndrome, genetic risk and deterioration syndrome (history of psychotic disorder in a first degree relative or schizotypal personality, and a 30% reduction in Global Assessment of Functioning Scale results in the past year), and attenuated psychosis syndrome (attenuated psychotic symptoms in the past year that have been present at least once a week in the last month and have not reached a psychotic level). The Structured Clinical Interview for DSM-5 is a semi-structured interview used to diagnose disorders (including psychotic disorders) according to DSM-5 criteria.

After these interviews, 60 of the 133 individuals were determined to meet ARMS criteria and the remaining 73 were included as healthy controls.

### Speech elicitation protocol

Two protocols were applied and audiovisual files were recorded using mobile phones. The first was an overview of the SIPS, during which participants were also asked to speak freely about their childhood and their relationship with their parents (subjective overview). The second, based on the experimental paradigm of Mota,^29^,^39^,^40^ requested oral memory reports of a recent or old dream and short-term memory reports based on three positively affective pictures – a baby, a puppy, and a dessert. Participants who could not remember any recent dreams were asked to report a previous dream they could recall.

Both protocols were recorded using a mobile phone camera supported on a table between the interviewer and the participant. The camera faced the participant frontally. The recordings were made during the day and with the lights on in rooms with windows.

After recording, the video was immediately stored in a secure cloud service and deleted from the phone. Protection was guaranteed through current encryption protocols in the backend database and remote secure socket layer communications according to Brazilian data protection compliance standards (https://www.lgpdbrasil.com.br).

### Media preprocessing

Original media was recorded on the interviewers’ smartphones. The recordings were in mp4 format (H.264 High Profile video codec and MPEG-4 AAC audio codec) with a resolution of 640 x 352 pixels at 25 frames per second (1,109 kbps bit rate). The audio was recorded in stereo with a 48,000 Hz sample rate (320 kbps bitrate).

FFmpeg software (v4.2.4) was used to extract the audio from the videos to FLAC format. We produced transcripts using OpenAI’s Whisper^41^ software and then manually corrected minor errors through a post-editing: a native speaker of Brazilian Portuguese with an undergraduate degree listened to all audio files and checked for transcription discrepancies, modifying them accordingly.

### Natural language processing

For a global assessment and to address the heterogeneity of ARMS speech, we chose complementary NLP methods to examine structural and semantic properties.^14^ Previous studies have found different types of speech changes in ARMS, and several methods have combined multiple speech facets.^4^ We introduce RQA and graph centrality techniques, which consider whole-speech relationships among the entire set of words (Supplementary Material S1).

Concerning structure, we analyzed verbosity (the number of words), part-of-speech tagging, and graph embeddings (e.g., the centrality, density, size, and number of SCC). Concerning semantics, we examined average sentiment polarity and semantic coherence (classic measures and RQA).

#### Preprocessing

Text preprocessing includes removing irrelevant characters (e.g., spaces and punctuation whenever adequate) and performing transformations to obtain unique tokens (e.g., removing accentuation and upper casing).

#### Grammatical classes (part-of-speech tagging)

Part-of-speech tagging was performed using the UDPipe parser for Portuguese.^42^ In part-of-speech tagging, phrases are parsed and words are labeled according to their syntactic function. The relative frequency of 15 classes were used as features: adjectives, adpositions, adverbs, auxiliary words, coordinating conjunctions, determiners, interjections, nouns, numerals, particles, pronouns, proper nouns, punctuation, subordinating conjunctions, symbols (special non-alphanumeric characters), and verbs. A full definition for each class is available online (https://universaldependencies.org/u/pos/index.html).

#### Graph embeddings (word repetition graphs)

**Figure 1:**
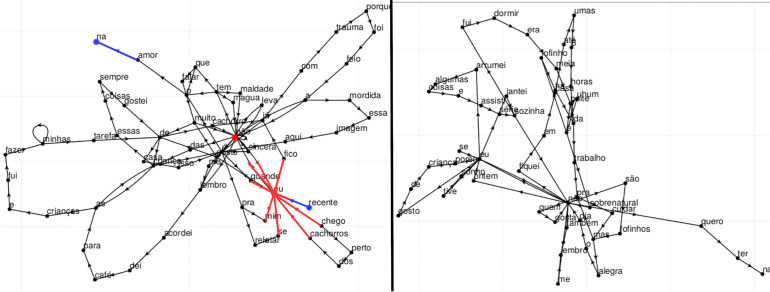
Graphs with low (97th quantile, left panel) and high (third quantile, right panel) average centrality_15_ from our sample. Nodes with small centrality (blue) are located at the extremities. Nodes with large centrality (red) are more likely to act as a bridge in the shortest path between two other nodes (betweenness centrality) and possess more edges (degree centrality).

Graph embeddings were produced using word repetitions and calculated connectivity measures. Connectivity is often reported to be associated with psychosis and is generally addressed through the count and size of SCC.^18^,^43^ We also used centrality measures to assess the profile of network connections. Centrality assigns a value to a node based on its connections to other nodes, including non-neighboring ones (“friends-of-friends”). We calculated three metrics of centrality: closeness centrality (the average length of the shortest path between the node and all other nodes in the graph), betweenness centrality (the number of times a node acts as a bridge along the shortest path between two other nodes), and eigenvector centrality (more connections with highly connected nodes yields a higher score).^44^-^46^ See Figure 1 and Supplementary Material S1 for details.

#### Sentiments

Sentiment analysis was used in conjunction with the National Research Council-Canada Portuguese corpus.^47^ This technique summarizes the polarity of words based on a corpus of previously labeled texts.^48^ It has been hypothesized that features generated from sentiment analysis can effectively distinguish between individuals with first-episode psychosis and healthy controls.^49^ To obtain overall valence, we took the average of all negative (valued as -1) and positive (+1) words in each text.

#### Semantic trajectories

In latent semantic analysis, words are mapped to real-valued vectors in a previously constructed semantic space. The distance between these vector representations is associated with the semantic similarity between words.^21^

The space can be constructed, for instance, by checking the relative co-occurrence of words in a corpus of documents. We used semantic spaces from FastText,^50^ which involves more complex procedures to perform embeddings. We obtained the standard metrics described in the literature: the maximum and average distances between FOC and SOC.^13^

We also used RQA to study a set of metrics obtained from the overall trajectory. This technique is used to study global characteristics of complex trajectories in dynamical systems.^51^,^52^ Certain internal structures of this matrix reflect trajectory characteristics.^53^

A total of 14 RQA metrics from four groups were used: one of them quantifies all similar pairs (recurrence rate [RR]) relative to all words. The number of words between two similar words is associated with three features, called Poisson times: mean recurrence time, recurrence time entropy, and number of the most probable recurrence time. For instance, mean recurrence time/ number of the most probable recurrence time is the average number of states between recurring states.

Some sequences of words are similar to a single word, as if revolving around a unique theme. Four features are related to this pattern: laminarity (LAM), trapping time (TT), maximum length of vertical structures (V_max_), and entropy of vertical structures (VENTR). TT is the average length of such structures, while V_max_ and LAM are the proportion of these states in the entire trajectory. We can also obtain the Shannon VENTR or the sequence.

**Figure 2:**
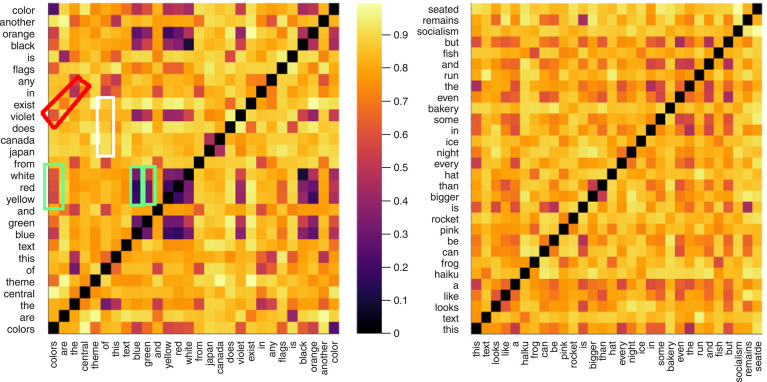
Distance matrix for a text about colors (left panel) and a text with random words (right panel) using cosine distance and FastText embeddings. Semantic distances range from 0 (very close, dark purple) to 1 (very far, light yellow). Patterns in the right panel resemble random noise, with no marked vertical, diagonal, or in-between structures. In the left panel, three green rectangles highlight vertical segments, showing similarity of themes in sequences of words. They relate the sequence “yellow, red, white” (12th-14th words) to three other states (first: “colors”; ninth: “blue”; 10th: “green”). The red diagonal shows a high order coherence (18th order): “colors are the” and “violet exist in” (x_1,19_, x_2,20_, and x_3,21_). The white segment shows a five-word (x_6,15_ to x_6,21_) interval between recurrences (Poisson time).

Diagonals describe similarities between sequences separated by non-similar words, including FOC, SOC, and higher-order coherences. Six features related to these patterns are described in the Supplementary Material S1. Figure 2 shows examples with heat map visualizations.

#### Sensitivity analysis: verbosity and demographics

Since some features might be affected by the total number of words uttered (verbosity), we used original texts and windowed versions. The windowing procedure consists of calculating the characteristics for subsets of text. For instance, the four-word excerpt “Green ideas sleep furiously” with a window size of two yields the three subsets: (“green,” “ideas”), (“ideas,” “sleep”), and (“sleep,” “furiously”). The average value of the subsets is then calculated.

Whenever non-windowed excerpts were used, we calculated Spearman partial correlations including verbosity (total transcript length in words) as a covariate if NLP features were found to be significantly associated with symptoms.

### Psychometric analysis

To assess the psychometric properties of SIPS, we used bifactorial models to extract a general latent trait (g) and orthogonal components (F_n_). It was expected that exploratory factor analysis would capture an instrument’s internal structure, e.g., shared variance among SIPS items and its domains: positive, negative, disorganized, and general symptoms. Models were evaluated according to squared errors (root mean square residuals and root mean square error of approximation). Trait uniqueness was evaluated using general factor saturation: hierarchical omega represents the proportion of total-score variance that is due to the single factor.^54^ The general latent score (g) and factor scores (F_n_) of each individual were used to investigate correlations with independent measures (e.g., speech features).

### Statistical inference

We analyzed Spearman’s correlations between each SIPS factor and NLP feature. We used regression models to assess linear relationships between NLP features and the probability of ARMS in the sample. We assumed a binomial distribution for the response variable and used a logit link function (logistic regression). We report parameter values and p-values for covariates. For binomial models, we present Tjur adjusted R^2^ value and the area under the receiver operating characteristic curve (AUROC) based on predicted probabilities and bootstrapped standard error.

Due to the large number of statistical tests, the Benjamini-Hochberg^55^ procedure was used groupwise to account for false discovery rate when looking for correlations between SIPS factors/ARMS and NLP features.

We performed tests with five graph properties (density, three types of centrality, and the size of largest SCC), four classic coherence measures (minimum, maximum, median, and mean FOC), one general coherence measure (RR), six coherence measures associated with regular intervals (diagonals), four coherences measures associated with sequences (vertical lines) and three measures associated with recurrence time, 15 part-of-speech classes, and one measure of verbosity (number of words).

### Machine learning models

Machine learning models were used to check whether NLP features were linked to ARMS status. Such models can identify non-linear patterns that differ among groups.

Boosting methods are a set of algorithms capable of leveraging weak classifiers (a machine learning classifier that is just slightly better than random guessing) into strong classifiers by training them sequentially and contributively.^56^ During the training of a boosting machine-learning model, the input data is modified according to performance in the previous step, with higher weights assigned to incorrectly classified samples, while correctly classified samples are given lesser importance. Thus, harder to classify examples are given greater influence during training. The final predictor’s decision is the outcome of the weighted decision regarding number of trained weak learners.^57^

Three different machine-learning algorithms were employed during this step: gradient boosting machines, AdaBoost, and random forests. Due to its capacity to determine the importance of a feature during training, gradient boosting machines were employed to search for the best set of features by utilizing the value ascribed to a feature depending on how many times it was used in model training, which is called feature importance. The final model is a combination of AdaBoost with random forests as weak learners, since this combination of ensemble methods has shown to be robust to overfitting and to lower error rates.^58^-^62^ Details about each model type and the training procedures are provided in the Supplementary Material S1.

### Software

The following software was used in the analysis: R programming language and computing environment (v4.2.2), including the packages stats (v4.2.2) udpipe (v0.8.9), syuzhet (v1.0.6), and psych (v2.2.5); Julia language (v1.7.2), including the packages DynamicalSystems (v2.3.2), TextGraphs.jl (v0.8.0), and SemanticTrajectories.jl (v0.1.0.); Python (v.3.8.10), including the packages scikit-learn (v1.2.2); and LightGBM (v3.3.2).

The open-source packages TextGraphs.jl and SemanticTrajectories.jl are user-developed projects that implement procedures unprecedented in literature. They can be found in official registries of Julia.^63^,^64^

### Ethics statement

The project was approved by the University of São Paulo ethics review board. All participants provided informed consent.

## Results

The demographic and clinical characteristics of the sample are shown in Table 1. The sample was 34% male and the mean age was 27 years.

### Factor analysis

A three-factor solution provided an adequate fit (root mean square error of approximation = 0.079, 95%CI 0.061-0.097; α = 0.84; ω_hierarch._ = 0.46) for the 19 items evaluating psychosis-risk symptoms (SIPS).

Latent factors reflected original domains (negative, positive, disorganized, and general) (Table 2). Factor 3 (F_3_) loadings were restricted to negative items (1,3,4,5; F_3_ correlation with the sum of negative items ρ = 0.606, p < 0.001), while factor 2 (F_2_) loadings included all positive items (1 to 5; sum of positive items correlation: ρ = 0.927, p < 0.001), along with two disorganized, one general, and one negative symptom. Factor 1 (F_1_) loadings were distributed between general (ρ = 0.739), disorganized (ρ = 0.471), and negative (ρ = 0.455) items (all p < 0.001). Negative item 5 (decreased ideational richness) had loadings from all three factors.

### Association between natural language processing assessment and psychotic symptoms

A summary of significant results corrected for multiple comparisons can be found in Table 3.

#### General factor

The general factor obtained from SIPS analysis was correlated with semantics and structure of speech. The main semantic markers were features identifying sequences of similar words in five-windowed texts: TT, V_max_ of the sequence, and LAM. In addition, VENTR, mean recurrence time (five-window), and trend (30-window) presented marked correlations (p < 0.050), but not after adjustment.

Concerning structure, there was a significant inverse correlation between mean betweenness centrality (15-window) and the general factor. The same was true for the size of the largest SCC, although not after correction. Individually, the mean betweenness centrality_15_ was correlated with SIPS negative items 1 (social anhedonia), 3 (expression of emotion), and 6 (occupational functioning).

#### Mixed symptoms (negative, disorganized, general)

The mixed latent F_1_ (including negative, disorganized, and general traits) was correlated with (15-windowed) TT, LAM, VENTR, and overall RR. Non-windowed entropy and five-windowed overall RR were also correlated with this factor. The sixth negative SIPS item (occupational functioning), along with general item 2 (dysphoric mood), 4 (impaired tolerance to normal stress), and disorganized third (trouble with focus and attention) seemed to drive this association.

Regarding individual items, TT_15_ and VENTR were related to the sixth negative item and the second and fourth general items. VENTR was correlated with the third disorganized item. VENTR_15_ and RR_15_ were correlated with the sixth negative and fourth general items. LAM_15_ was significantly associated only with the second and fourth general items. RR_5_ was associated with the fourth general item.

#### Negative symptoms

Latent F_3_ (mostly negative items) was correlated with (five-windowed) TT, V_max_, and mean recurrence time.

The first (social anhedonia), third (expression of emotion), and fourth (experience of emotions and self) negative items were significantly associated with NLP features. TT_5_ was related to the first, third, and fourth items. Vmax_5_ was only associated with the third and fourth items, and MRT_5_ was only associated with the first item.

#### Positive symptoms

F_2_ (mixed and all positive) was correlated with adjective use (ρ = 0.281; p < 0.001). Individually, it correlated with individual items 1 (unusual thought content) and 4 (perceptual abnormalities/hallucinations).

### Multiple tests and verbosity adjustments

Several NLP features were significantly correlated with psychotic symptoms at first, but not after rigorous adjustments for multiple testing and verbosity. The maximum FOC was inversely correlated with the negative factor. The size of the largest connected component in 30-windowed texts and TREND_30_ was inversely correlated with the general factor. The size of the largest SCC_30_ was also correlated with the positive trait (F_2_). Verb usage was positively correlated with the general factor. The negative factor was associated with verb and interjection use. Adjective use, SCC_30_, graph density_1_, and determinism_30_ differed between the ARMS group and controls in Wilcoxon tests.

### At-risk mental states status and natural language processing features

Adjective use, determinism (DET_30_), and MRT_5_ differed between the ARMS and control groups according to a multivariate linear model (t-statistic test for regression parameters, H_0_ : θ = 0), with binomially distributed outcomes and logistic link function (Tjur model R^2^: 0.108; bootstrapped AUROC: 70.72%; standard error = 4.5%).

The results of the machine learning model are shown in Table 4, including the mean, SD, maximum and minimum values after the validation procedure with 100 repeated holdouts. According to the results, NLP features yielded good predictions when distinguishing between ARMS and control status (AUROC = 0.93; balanced accuracy = 0.86; F_1_-score = 0.85; sensitivity = 0.84; specificity = 0.88).

**Figure 3:**
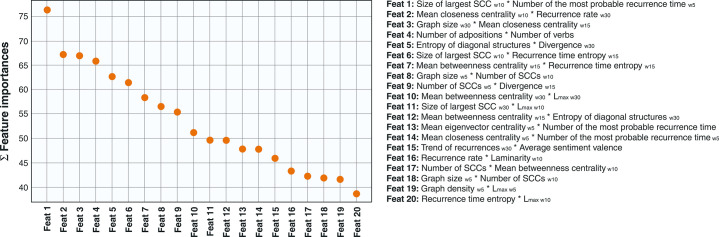
Top 20 features found through the feature permutation method. The values indicate the sum importance of each feature after 30 different training loops with 100 permutations of each feature. Feat = feature; SCC = strongly connected components.

The features were obtained after pairwise interaction and selection steps. Thus, except for DET_30_, every selected feature was a polynomial combination of the originals. Figure 3 shows the 20 most important features after assessing and summing their importance.

## Discussion

Factor analysis of the SIPS scores resulted in three symptom dimensions. Correlating them with NLP features, the positive symptom dimension was significantly associated with adjective use. Negative and mixed/non-positive symptoms were correlated with features related to a single topic (vertical structures) and the number of unrelated words between similar words (recurrence times). Negative symptoms were correlated with TT, V_max_ of vertical structure, and mean recurrence time. Mixed/non-positive symptoms were correlated with LAM, VENTR, and RR. Our machine learning model reached a balanced accuracy of 86% in distinguishing between ARMS and control status (AUROC 93%). Four of the most important features used by the model were: feature 1, largest SCC at a window size of 10 weighted by the frequency of the most frequent recurrence time; feature 2, mean closeness centrality weighted by RR; feature 3, mean closeness centrality weighted by graph size at a window of size 30; and feature 4, adposition frequency weighted by verb frequency. Our results are yet another step toward linking NLP features to clinically assessed psychopathology, reinforcing the potential of automatically generated NLP features to enhance clinical practice, either in ARMS screening or in psychometric assessment of language and thought disorders.

Fortuitously, centrality and RQA metrics debuted as outstanding NLP-based sources of information compared to standard metrics. Further studies are needed to reproduce these findings. If confirmed, their successful performance of can be attributed to the whole-speech approach. Centralities seem to reflect connectivity by capturing global network topology. Similarly, RQA considers varied structures in the entire text, as compared to measures that consider homogeneous (ergodic) trajectories, such as the average, or measures that consider extreme points, such as maximum and minimum. The software we used is open-source and is available through plug and play libraries, making it easy for other groups to benefit from this asset and replicate our results.

### A blueprint for psychometric assessment with natural language processing

Besides enhanced precision/information for analysis, the vocabulary brought forward by RQA reveals new features about psychotic speech. We outline some of them in Table 5, in which some important features in the model can be linked to items in the Thought, Language, and Communication and Thought Disorder Index.^65^ A blueprint connecting each clinical characteristic and efficient methods of automated processing seems near.

RQA acquisition assembles opposing characteristics in psychotic speech because it can capture details of different sections and structures simultaneously. This is useful, since psychotic speech shows drifting sections as well as unusually long connected trails (e.g., perseveration).^7^ RQA vertical structures (e.g., LAM, TT, size of the longest sequence) characterize these sections through interconnected words revolving around a single topic. We link these features to the clinical construct of perseveration. The mixed symptom dimension of psychosis, with negative, disorganized, and general symptoms, was correlated with snippets consisting of consecutive similar words, i.e., frequency, average/maximum size, heterogeneity, and the average number of unrelated words between such snippets.

Additionally, RQA Poisson time (e.g., intervals between recurrences) targets incoherence in a different manner by grasping sequences of incoherent text instead of measuring average coherence or abrupt breaches (maximum and minimum). This group can be linked with drifting themes in circumstantiality and distractible speech.

RQA diagonals concern the presence of regular intervals (e.g., SOC, third order coherence, etc.). Although not significantly associated with psychotic symptoms, they provided information for the machine learning ensemble ARMS classifier.

Changes in adjective frequency have been reported in studies of English-speaking patients,^26^,^66^ and we found similar results in Portuguese language part-of-speech tagging. Nevertheless, adjective frequency was positively correlated with positive symptoms, and the ARMS group used more adjectives than the control group, whereas previous studies indicated the opposite. Further studies could determine whether this difference is due to the language or to other study settings. Nevertheless, practitioners may find important clues in speech by examining its grammar. Regardless of the baseline frequencies in each language, individuals with ARMS seem to deviate from the norm, often through excessive (or reduced) pronoun and adjective use.

Since there is a consensus about clinical manifestations of emotional impairment among specialists, we expected to find a relationship between sentiments expressed in speech and psychotic traits. However, we did not. This aligns with Mota et al.,^39^ who failed to find a direct association between emotional expression with symptomatology, although they did find an indirect association through speech connectedness.

To analyze speech coherence, we used FastText semantic spaces.^67^ In accordance with other approaches in the literature, this method provided measures of semantic similarity that were highly correlated with SIPS factors. A systematic review by de Boer et al.^68^ pointed out that most studies (n=17) have used latent semantic analysis to calculate semantic spaces. Other less frequent methods included latent semantic indexing, singular value decomposition, and word2vec. Boer et al. recommended that researchers focus on newer methods. We report relevant findings with FastText, which uses subword information: each word is represented as a set of character n-gram rather than a fundamental unit of analysis. This provides small execution times, and word vectors are currently available for 157 languages, which makes it a valuable resource for studying non-English speaking populations.

Several features based on graph embeddings (including density, centralities, number of SCCs, and size of the largest SCC) were correlated with negative, disorganized, and general symptoms. However, non-windowed measures were no longer significant when partial correlations included verbosity, and most windowed correlations did not survive p-value adjustment. Accordingly, verbosity has been empirically correlated with properties such as the size of the largest component in graphs and minimum FOC.^13^,^28^ We hypothesize that some of the structural characteristics found in psychotic speech may actually be a side product of proneness to elaborate speech.

Centrality measures evaluate the position of a given node according to all network connections. This can involve only nearby structures (“friends”), as in degree centrality, or indirectly connected nodes (“friends-of-friends”) and so forth (“friends-of-friends-of-friends”). For instance, betweenness centrality quantifies the number of times a node acts as a bridge along the shortest path between every pair of nodes. They are powerful methods of evaluating connectivity in networks.^69^-^71^ The literature has already identified network connectivity as an important speech feature for psychometric assessment. The number and size of SCCs are generally used for this purpose. Further research could determine the type of information derived from centrality analysis.

### Natural language processing and at-risk mental state screening

The early detection and prevention of mental disorders has gained much attention. Public policies that actively promote mental health are becoming more common as the population gains more insight into the burden of mental disorders. Because ARMS screening is one of the most well-studied preventive paradigms, there is a corresponding search for a reliable clinical marker of ARMS status.^14^,^68^

Independent systematic reviews^4^,^10^,^68^ have suggested that NLP methods are producing consistently valid and accurate results. However, the data have mostly been derived from studies with limited sample sizes and non-epidemiological designs. We present positive psychosis screening results, capitalizing on an epidemiological study with a large general population sample from São Paulo, South America’s most populous city. It is among the first ARMS studies undertaken in the Portuguese language, reproducing earlier results in a more generalizable setting.

Our findings show potentially important practical implications. Health care systems are generally unable to provide specialized care for each medical specialty in under-resourced settings.^72^ In such scenarios, scalable technology to detect mental illness would be especially interesting for public health screening purposes.^73^ The use of automated tools for capturing and processing behavior is a recent development in psychometrics,^59^,^74^ following technological advances and feasible hardware and software costs. This innovation provides objective measures of behavioral cues for screening, diagnosis, follow-up, and prognosis.^75^ NLP findings should expand the current corpus of knowledge to further enable automatic detection.

Furthermore, one of the greatest difficulties in psychiatry is the inter-reliability of assessments between professionals. Subjective human assessment precludes standardized metrics, such as those used to measure natural phenomena in physics. Despite efforts to increase assessment homogeneity through training, psychometric scores are generally influenced by observer bias when prompting questions and interpreting responses. Computerized methods overcome this problem through automated processes, which use standardized procedures for each individual.^76^,^77^ In recent decades, advances in hardware capabilities and computational theory have allowed for explanatory frameworks based on symbolic processing and statistical models. In this context, readily available wearables and smartphones can become part of the practitioner’s toolset.

Our study has several limitations. One of those is discourse effort. Results require a significant amount of preprocessing and involve several sensitive points. They also depend on the availability of corpora in the studied language. We must aim at validation across different cultures through adequate sampling procedures to make findings generalizable.

The language patterns used to distinguish the ARMS and healthy control groups may also reflect context-related features. Individuals with ARMS may present certain speech characteristics in different situations, for instance, during clinical assessment, when recalling dreams from memory, or when describing pictures.

Psychologists and psychiatrists have made many attempts to improve reliability, often facing problems such as reductionist taxonomy. The idea of an unbiased computer performing behavioral assessment is compelling at first sight, although there are pitfalls. When using complex algorithms in clinical decision-making, we must be especially aware of the interpretability and generalizability of the results.

We presented findings that expand current knowledge of NLP analysis in mental illness. We highlight three main issues, namely the association of NLP features with psychopathology, the good accuracy of our model in predicting ARMS status, and the use of a populational sample. We also point out the novel use of RQA in ARMS assessment.

Our results are yet another step towards automatic detection of individuals at risk for psychosis. NLP provides a scalable tool that could fuel public health initiatives to screen the population and promote early intervention. Future studies should address the intercultural validity of the results and should enroll larger samples to ensure generalizability.

Regarding NLP, we suggest two topics that could be addressed in future studies, namely the setting (e.g., medical room), and interaction with interlocutors and groups. The environment itself modulates verbal expression and little is known about this variable, e.g. how it varies according to comfort level (clinical facilities vs. the patient’s home), ambient noise, and the presence of other people. Dyadic and collective speech modeling may also help clarify the relationship between speech and mental illness, and it has not yet been covered in NLP research. Many psychotic features involve social behavior (e.g., negative symptoms), and some speech features could be more informative and more evident during conversation.

## Disclosure

FA has provided consulting services and developed technology for private companies. NBM works at Mobile Brain, an Education and Health Tech startup, and has been a consultant to Boehringer Ingelheim. JMG works at Mobile Brain and has provided consulting for developing machine learning models for private companies. The other authors report no conflicts of interest.

## Figures and Tables

**Table 1:** Demographic and clinical characteristics of the sample

Characteristic	Full sample (n=133)	ARMS status	
		No (n=73)	Yes (n=60)
Age (mean ± SD)	27±5	27±5	27±4
Sex			
Female	87 (67.0)	49 (68.0)	38 (66.0)
Male	43 (33.0)	22 (32.0)	17 (34.0)
Ethnicity			
White	67 (52.0)	35 (49.0)	32 (55.0)
Black	25 (19.0)	16 (22.0)	9 (16.0)
Mixed	37 (28.0)	20 (28.0)	17 (29.0)
Marital status			
Single	99 (76.0)	53 (74.0)	46 (79.0)
Married	16 (12.0)	10 (14.0)	6 (10.0)
Divorced	4 (3.1)	4 (5.6)	0 (0.0)
Stable union	11 (8.5)	5 (6.9)	6 (10.0)
Has children	34 (26.0)	17 (24.0)	17 (29.0)
Number of children			
0	99 (74.0)	56 (77.0)	43 (72.0)
1	21 (16.0)	12 (16.0)	9 (15.0)
2	11 (8.3)	3 (4.1)	8 (13.0)
4	1 (0.8)	1 (1.4)	0 (0.0)
Employed	87 (67.0)	49 (68.0)	38 (66.0)
Education level			
Grade-school (complete)	2 (1.5)	0 (0.0)	2 (3.4)
High School	5 (3.8)	1 (1.4)	4 (6.9)
High school (complete)	36 (28.0)	19 (26.0)	17 (29.0)
Undergraduate	33 (25.0)	20 (28.0)	13 (22.0)
Undergraduate (complete)	41 (32.0)	24 (33.0)	17 (29.0)
Graduate	4 (3.1)	3 (4.2)	1 (1.7)
Graduate (complete)	8 (6.2)	4 (5.6)	4 (6.9)

Data presented as n (%).

ARMS = at-risk mental states.

**Table 2:** Item loadings by factor

Factors	Items loaded by domain			
	Positive	Negative	General	Disorganized
Factor 1: mixed, non-positive	-	2, inverse 5, 6	1, 2, 4	3, 4
Factor 2: mixed, all positive	1 to 5	4, 5	3	1, 2, 3
Factor 3: negative	Inverse 3	1, 3, 4, 5	-	-

**Table 3:** Significant correlations between NLP and SIPS traits

			Factor					
	g		1 (mixed, non-positives)		3 (negative)		2 (positive)	
Feature	ρ	p-value	ρ	p-value	ρ	p-value	ρ	p-value
TT_5_	0.211	0.015	-	-	0.220	0.011	-	-
TT_15_	-	-	0.245	0.005	-	-	-	-
Vmax_5_	0.201	0.021	-	-	0.175	0.044	-	-
LAM_5_	0.199	0.022	-	-	-	-	-	-
LAM_15_	-	-	0.212	0.014	-	-	-	-
VENTR	-	-	0.266	0.002	-	-	-	-
VENTR_15_	-	-	0.231	0.007	-	-	-	-
RR_15_	-	-	0.232	0.007	-	-	-	-
MRT_5_	-	-	-	-	0.208	0.016	-	-
Average betweenness centrality_15_	-0.238	0.006	-	-	-	-	-	-
Use of adjectives	-	-	-	-	-	-	0.281	0.001

Subscript text indicates window-size whenever windowed text was used.

LAM = laminarity; MRT = mean recurrence time; NLP = natural language processing; RR = recurrence rate; SIPS = Structured Interview for Psychosis-Risk Syndromes; TT = trapping time; V = maximum length of vertical structures; VENTR = entropy of vertical structures.

**Table 4:** Machine learning model performance

	Mean ± SD	Maximum	Minimum
Factor 1-score	85±6	100.00	64.52
Specificity	88±7	100.00	68.18
Sensitivity	84±8	100.00	55.56
Balanced accuracy	86±5	100.00	70.96
AUROC	93±3	100.00	85.86

Data presented as percentage, unless otherwise specified.

AUROC = area under the receiver operating characteristic curve.

**Table 5:** Thought and speech changes and NLP techniques designed for assessing them

	Poverty of speech		Verbosity	Blocking	Incoherence, derailment, and unexpected themes			Perseveration	Mimic behavior
TLC	Poverty of speech	Poverty of speech content	Pressure of Speech	Blocking	Derailment	Incoherence	Self-reference	Perseveration	Echolalia
TDI	Impoverishment of thought and speech	Poverty of speech	-	-	Looseness	-	-	Perseveration	-
NLP feature	Word repetition and graph embeddings	Grammatical tagging	Verbosity (sentence size, total number of words per time unit)	Duration and number of pauses	Semantic distances in words and phrases			Vertical structures in RQA of semantic embeddings†	‡
	Circumstantiality and related				Clanging	Logic	Misplaced words/neologisms	Odd structure	
TLC	Circumstantiality	Distractibility	Loss of goal	Tangentially	Clanging	Illogicality	Word approximations	Stilted speech	
TDI	-	Distractible speech	Weakening of goal	-	-	Peculiar logic	Peculiar word use	Peculiar sentence construction	
NLP feature	Poisson times in RQA of semantic embeddings‡			Slope of sequence of cosine distances^22^	‡	‡	‡	‡	

NLP = natural language processing; TLC = Thought, Language and Communication; TDI = Thought Disorder Index.

† Features introduced with recurrence quantification analysis.

‡ Items still unaddressed by NLP methods.
