# Supplementary material for: Natural language processing in at-risk mental states: enhancing the assessment of thought disorders and psychotic traits with semantic dynamics and graph theory
Source: Braz J Psychiatry. 2024 Nov 25;46:e20233419. doi: 10.47626/1516-4446-2023-3419 (PMC11773321; doi:10.47626/1516-4446-2023-3419)
Supplement: Supplementary file 1 [file bjp-46-e20233419-suppl1.pdf]

**Supplementary Material S1**

## Natural language processing and machine learning methods

### Semantics

*Semantic coherence with recurrence quantification analysis of latent space embeddings*

We introduce recurrence quantification analysis as a method of semantic coherence analysis. In recurrence quantification analysis, a trajectory with *n* states is analyzed through a matrix *n* * *n*. This matrix has 0-valued entries when two states are far apart and 1-valued entries when they are close. For instance, if entry $x_{1,4}$ has a value of 1, then the first and fourth states are close (e.g., distance smaller than threshold).

Cosine distance is widely used to evaluate distances in semantic embeddings. Unfortunately, it is not a metric in the strict sense. That is, it does not satisfy properties such as triangle inequality (e.g., the sum of any two sides of a triangle is greater than or equal to the third side). To circumvent this problem and obtain lower-dimensional trajectories for recurrence quantification analysis, we use t-distributed stochastic neighbor embedding to generate 2D projections from the original cosine based distance matrices. The full procedure consists of first transforming words into a sequence of 300-dimensional vectors using latent space embeddings (e.g., FastText, BERT) and then calculating pairwise cosine distances between each pair of words and using t-distributed stochastic neighbor embedding to produce a lower-dimension version of the trajectory.

Recurrence quantification analysis was applied considering the Euclidean distance as the metric and the average distance value as a threshold. In addition to the full texts, we examined windowed subsets of size 5, 10, 15 and 30. The resulting features are grouped into 4 types, described as follows.

**The recurrence rate** is the relative frequency of states in which the trajectory returns to previously visited positions, akin to a general measure of coherence, considering all similar word pairs in the text.

**Recurrence time** measures how many intermediary states exist between an arbitrary state and a recurrence, i.e., how long it takes for similarities to occur. They are related to the Poincaré recurrence theorem, which proves that certain systems will eventually return to a state arbitrarily close to their initial state. Poincaré recurrence time is the length of time elapsed until a recurrence. A total of 3 features assess this: mean recurrence time, recurrence time entropy, and the number of the most probable recurrence time. The mean recurrence time is the average interval between similar words. Recurrence time entropy is used to determine whether recurrence times are heterogeneous (i.e., appearing at different frequencies). The number of the most probable recurrence time indicates how many times recurrences exist considering the most frequent recurrence time.

**Vertical lines** relate a given word and the next n-words. For instance, a vertical line formed by points $x_{1,2}$, $x_{1,3}$ and $x_{1,4}$ indicates a small semantic distance between the first and the next 3 consecutive words. A total of 4 features are associated with vertical lines: trapping time, laminarity, maximum length, and entropy of vertical structures.

Trapping time is the average size of these consecutive coherent snippets. Laminarity is the percentage of recurrence points that form vertical lines, i.e., how many coherent pairs belong to a consecutive sequence as compared to random coherent pairs. The maximum length indicates the longest sequence. The Shannon entropy of vertical structures indicates whether coherent sequences appear at any size and whether some sequence sizes are more frequent than others.

**Diagonals** suggest regular intervals with different delays. Each diagonal contains sequences of distances between words considering different step windows. For instance, the diagonal immediately next to the identity (main diagonal, line of identity) is called the superdiagonal (or 1-diagonal) and contains all first order coherences, while the 2-diagonal contains all second order coherences.

We studied 6 features from diagonals. The average length of diagonal structures represents the average size of coherent snippets of any order (e.g. first, second, and third order coherences). Determinism is the ratio between diagonals (count weighted by length) and total recurrence, i.e., how often coherent speech snippets with regular intervals (e.g., first and second order coherence sequences) appear compared to random coherent word pairs. The maximum diagonal length and its inverse, divergence (or 1/maximum diagonal length), shows the largest snippet with coherence in regular intervals.

We can also calculate the Shannon entropy of the probability distribution of the diagonal line lengths. Texts with similar diagonal lengths for all intervals (first, second, and third order coherences) will have higher entropy (akin to a uniform distribution) while imbalances in the distribution of diagonal lengths will be associated with smaller values.

The trend is the slope of a linear regression that relates the density of recurrent points in the diagonals parallel to the line of identity and the distance between those diagonals and the line of identity. If the trend is positive, recurrence at large delays is more frequent than for close words. If negative, recurrence is more frequent at small delays. Zeroed values suggest that similar words occur regardless of time window.

**Structure**

*Connectivity and friends-of-friends (centrality)*

In graph theory, centrality assigns numbers or rankings to nodes within a graph corresponding to their network position. Centralities weigh the importance of each node according to its relative position and the length of several network paths. There are several strategies for quantifying it: degree centrality is defined as the number of links incident upon a node.

$$C_D(v) = \deg(v)$$

The closeness centrality (or closeness) of a node is the average length of the shortest path between the node and all other nodes in the graph. Closeness was defined as the reciprocal of farness.[1] For a vertex v, $F(v) = (\sum_u d(u,v)) \text{ where } d(u,v)$, where d(u, v) is the distance between vertices u and v. We generally use its normalized form, given by the previous formula divided by N − 1, where N represents the number of nodes in the graph.

$$C_{Closen.}(v) = F(v)^{-1} = \frac{N-1}{\sum_u d(u,v)}$$

Betweenness centrality quantifies the number of times a node acts as a bridge along the shortest path between two other nodes.

$$C_{Between.}(v) = \sum_{s \neq v \neq t \in V} \frac{\sigma_{st}(v)}{\sigma_{st}}$$

where σst is total number of shortest paths from node s to node t and σst(v) is the number of those paths that pass through v.[2]

Eigenvector centrality assigns relative scores to all nodes in the network based on the concept that connections to high-scoring nodes contribute more to the score of the node in question than equal connections to low-scoring nodes. Google's PageRank is closely associated with the normalized eigenvector centrality.[3]

## Boosting methods

The prediction in boosting is given by: $G(x) = sign(\Sigma_{m=1}^{M} \alpha_m G_m(x))$, where $G_m(x)$ is the prediction of the weak classifier on step *m* of the training procedure, with a learned weight $\alpha_m$ and *sign* being the signal function.

### *Gradient Boosting Machines*

Gradient boosting machines (GBMs)[4,5] use the gradient descent method to set the weights of weak classifiers, minimizing a loss function chosen before the beginning of the training[6] and correlating each weak classifier at every training step with the negative gradient of the chosen loss function.[6]

We used an implementation of GBMs called LightGBM,[7] which employs GBMs with decision trees as weak learners. LightGBM applies two different techniques to deal with the size of the training data and the number of features of each sample: gradient-based one-side sampling and exclusive feature bundling. The purpose of gradient-based one-side sampling is to reduce the amount of training data, retrieving information from the gradient descent algorithm to decide if a data sample is well trained or not (whether its gradient is small or large). This information helps the model maintain samples that are not well trained and to perform random sampling from well-trained instances. The gradient-based one-side sampling procedure helps the GBM pay more attention to instances that were not correctly classified in the previous step. Exclusive feature bundling is used to decrease the number of features seen during training by searching for attributes that can be mutually exclusive, aggregating them into a small number of exclusive bundles.

Because LightGBM uses decision trees as weak classifiers, it can retrieve information from the trained trees and determine the importance of each feature during training in two different ways: the number of times a single feature is used to split a tree ("split" importance type) and the mean of information gain one feature adds when used to split a tree node ("gain" importance type). In the present study, "split" importance was used to decide whether a feature would be used to train the classifier or not.

### *AdaBoost*

AdaBoost[8] is a boosting method that applies additive weights to a set of weak learners, minimizing exponential loss. Considering a dataset $D_1$, with $x_1$, $x_2 \ldots x_n$ as its samples and the weak learners $G_1, G_2 \ldots G_n$, the AdaBoost algorithm attempts to create new datasets $D_2$, $D_3 \ldots D_n$ where samples misclassified by the base learners stand out. The base learner $G_1$ is trained with the original dataset ($D_1$), and weight $\alpha_1$ is determined by minimizing the loss function of the entire dataset at this step. This weight is later used to create every new sample $x_i$ of the next dataset ($D_2$) in distinct ways: $\exp(-\alpha_1)$ if $x_i$ was correctly classified or $\exp(\alpha_1)$ if $x_i$ was misclassified.[9] Hence, newly created datasets give more importance to previous misclassifications, helping the model correct previous mistakes.

### *Random forests*

The random forest method is an ensemble learning classification algorithm that improves decision trees by decorrelating them.[10] Multiple trees are built by repeatedly pulling samples from the original dataset (bootstrapping) with a number of different random features selected to create each tree. Selecting a subset of random forests prevents one strong feature from always being used as the first splitting node (root), which would result in similar, correlated trees. By creating different decision trees, the random

forest method assures a more flexible final classifier that can better generalize and yield fewer test errors.[8,10]

*Training*

When training a classifier, the interaction between pairs of features can be a good candidate for explaining variation in response variables.[11] NLP analysis elicited 153 features that were used to produce 11,781 new features with polynomial combinations, resulting in 11,934 features. The feature interaction step generates many features that can be used to distinguish study arm and control groups. We selected the best features by utilizing LightGBM's importance attributes feature.

To find these features, we trained 100 different LightGBM classifiers on 100 different training and testing splits (70% training, 30% testing). While training these classifiers, we determined the importance of each feature, summing them. After 100 training loops, each feature had a summed importance resulting from how many times they were used in each model that had been trained on a different subset of the data. These features were ranked based on the summed importance and we selected the top 0.5%, resulting in 62 attributes, which were reduced to 60 after removing those with a Spearman's correlation > 99% between them. These final 60 features were used to train our classifier.

To train the model, the Python scikit-learn library[12] was used to create an AdaBoost model with a random forest as the weak learner. The hyperparameters were 900 random forest estimators, each with 5 trees with a maximum depth of 1 (1 internal node + 2 leaf nodes). The maximum number of random features selected for each tree was $\log_2$; every other hyperparameter was the default set by scikit-learn.

To mitigate overfitting problems, leave-one-out (or Monte Carlo) cross-validation was employed, in which the data is split into training and testing sets (70% and 30%, respectively) in 100 different ways, with the final metric being the average of these 100 training loops.[13] These 100 different training and testing splits were set with different seeds from the splits performed during the feature selection step.

*Feature importance*

To facilitate interpretation of the predictions, we assessed the features by calculating their permutation importance. We did this by calculating the F1 score of the trained model on a test set and measured the score difference when one of feature was randomly permuted. The feature's importance was then determined by this score difference. Each feature was randomly changed 100 times, and we trained with 30 different train/test splits.

*Performance metrics*

The model was evaluated with five different metrics: F1 score, specificity, sensitivity, balanced accuracy, and receiver operating characteristic area under the curve. The F1 score represents the mean between precision and recall and is calculated as:

$$Precision = \frac{True\ Positive}{True\ Positive + False\ Positive},$$

$$Recall = \frac{True\ Positive}{True\ Positive + False\ Negative},$$

$$F1 = 2 * \left(\frac{Precision\ x\ Recall}{Precision + Recall}\right),$$

yielding a value that gives equal importance to precision and recall. Specificity and sensitivity evaluate the model's capacity to predict positive and negative outputs. Sensitivity is an alternative name for recall, and specificity is calculated as:

$$Specificity = \frac{True\ Negative}{True\ Negative + False\ Positive},$$

penalizing the score when false positives are predicted. These metrics are present in the balanced accuracy equation:

$$Balanced\ Accuracy = \frac{1}{2}\left(\frac{True\ Positive}{True\ Positive\ +\ False\ Negative} + \frac{True\ Negative}{True\ Negative\ +\ False\ Positive}\right),$$

Balanced accuracy is a metric well suited to evaluate models trained on imbalanced datasets.[14] Finally, the ROC curve is used to display the cost-benefit between the true positive rate (TPR) and the false positive rate (FPR), which are the number of positive samples correctly classified and the number of negative samples incorrectly predicted, respectively. It shows the rate of correct predictions at the cost of making mistakes in negative samples. TPR and FPR are calculated as follows:

$$TPR = \frac{True\ Positive}{True\ Positive + False\ Positive},$$

$$FPR = \frac{False\ Positive}{True\ Negative + False\ Positive} = 1 - Specificity.$$

## References

1. Bavelas A. Communication patterns in task oriented groups. J Acoust Soc Am. 1950;22:725-30.
2. Brandes U. A faster algorithm for betweenness centrality. J Math Sociol. 2001;25:163-77.
3. Gleich DF. PageRank beyond the web. SIAM Rev. 2015;57:321-63.
4. Friedman JH. Greedy function approximation: a gradient boosting machine. Ann Stat. 2001;29:1189-1232.
5. Friedman JH. Stochastic gradient boosting. Comput Stat Data Anal. 2002;38:367-378.
6. Natekin A, Knoll A. Gradient boosting machines, a tutorial. Front Neurorobot. 2013;7:21.
7. Ke Guolin, Meng Q, Finley T, Wang T, Chen W, Ma W, et al. LightGBM: a highly efficient gradient boosting decision tree. In: Proceedings of the 31st Conference on Neural Information Processing Systems (NIPS). Long Beach: NIPS; 2017.
8. Freund Y, Schapire RE. Experiments with a new boosting algorithm. In: Proceedings of the Thirteenth International Conference on International Conference on Machine Learning (ICML). San Francisco: ICML; 1996. p. 148-56.
9. Zhou Z-H. Ensemble methods: foundations and algorithms. Boca Raton: Taylor & Francis Group; 2012.
10. James G, Witten D, Hastie T, Tibshirani R. An introduction to statistical learning with applications in R. 2nd ed. Springer: New York; 2021.
11. Kuhn M, Johnson K. Feature engineering and selection: a practical approach for predictive models. Boca Raton: Taylor & Francis Group; 2019.
12. Pedregosa F, Varoquaux G, Gramfort A, Michel V, Thirion B, Grisel O, et al. Scikit-learn: machine learning in Python. J Mach Learn Res. 2011;12:2825-30.
13. Kuhn M, Johnson K. Applied predictive modeling. New York: Springer New York; 2013.
14. Brodersen KH, Ong CS, Stephan KE, Buhmann JM. The balanced accuracy and its posterior distribution. In: Proceedings of the 20th International Conference on Pattern Recognition (ICPR). Istanbul: ICPR; 2010. p. 3121-4.
